# A physician survey reveals differences in management of idiopathic pulmonary hemosiderosis

**DOI:** 10.1186/s13023-015-0319-5

**Published:** 2015-08-20

**Authors:** Chana I.C. Chin, Shirleen Loloyan Kohn, Thomas G. Keens, Monique F. Margetis, Roberta M. Kato

**Affiliations:** Division of Pulmonology, Children’s Hospital Los Angeles, Los Angeles, California; Department of Pediatrics, Keck School of Medicine, University of Southern California, Los Angeles, California

**Keywords:** Interstitial lung disease, Lung pathology, Pulmonary vascular disorders, Vasculitis, Idiopathic pulmonary hemosiderosis, Diffuse alveolar hemorrhage, Capilliritis, Lung biopsy

## Abstract

**Background:**

Idiopathic pulmonary hemosiderosis (IPH) is a rare disorder of unknown etiology characterized by chronic pulmonary hemorrhage and presents with a triad of anemia, hemoptysis and pulmonary infiltrates. IPH is a diagnosis of exclusion with a variable and disparate clinical course. Despite existing therapies, few children achieve full remission while others have recurrent hemorrhage, progressive lung damage, and premature death.

**Methods:**

We surveyed physicians who care for patients with IPH via a web-based survey to assess the most common practices. 88 providers responded, caring for 274 IPH patients from five continents.

**Results:**

63.3 % of respondents had patients that were initially misdiagnosed with anemia (60.0 %) or gastrointestinal bleed (18.2 %). Respondents varied in diagnostic tools used for evaluation. The key difference was in the use of lung biopsy (51.9 %) for diagnosis. Common medications respondents used for treatment at initial presentation and chronic maintenance therapy were corticosteroids (98.7 and 84.0 %, initial and chronic therapy respectively), hydroxychloroquine (33.3 and 64.0 %), azathioprine (8.0 and 37.3 %), and cyclophosphamide (4.0 and 16.0 %). There was agreement on the use of corticosteroids for exacerbation amongst all respondents. Reported deaths before adulthood occurred in 7.3 % of patients. We conclude that there were common features and specific variations in physician management of IPH. Respondents were divided on whether to perform lung biopsy for diagnosis.

**Conclusion:**

Despite the availability of various immunomodulators, corticosteroids remained the primary therapy. We speculate that the standardization of care for diffuse alveolar hemorrhage will improve patient outcomes.

## Background

Idiopathic pulmonary hemosiderosis (IPH) is a rare disorder characterized by diffuse alveolar hemorrhage. Its incidence is about 0.5–1 case per million [1, 2]. IPH classically presents with a triad of anemia, hemoptysis and diffuse pulmonary infiltrates. Some patients achieve full remission while others have recurrent hemorrhage, progressive lung damage and premature death [3]. IPH is more common in children than adults with patients often presenting prior to 10 years of age. However, the diagnosis can be delayed in young children who swallow their sputum and do not present with hemoptysis [3–7]. IPH is a diagnosis of exclusion and other etiologies such as autoimmune, cardiac, and infectious disease must be assessed first. Basic diagnostic studies can include hemoglobin content, reticulocyte count, chest radiography, bronchoalveolar lavage, chest CT, and lung biopsy. Evaluation for milk protein allergy (Heiner Syndrome) and celiac disease (Lane Hamilton Syndrome) can also be part of the diagnostic evaluation [8–13].

Though there is a consensus on the evaluation of childhood interstitial lung disease in children less than 2 years of age, there is no consensus on how to evaluate diffuse alveolar hemorrhage or utilize available therapies [14]. Treatment with corticosteroids is the most common approach, but other treatments, such as hydroxychloroquine (HCQ), azathioprine (AZA) and cyclophosphamide (CYC) have been proposed in small pediatric case studies and adult trials [3, 6, 7, 15–22]. Rare treatment options like intravenous immunoglobulin (IVIG), plasmapharesis, methotrexate (MTX), N-acetylcysteine, mycophenolate, 6-mercaptopurine and diet modification have also been suggested in case reports [3, 7, 10, 11, 19, 20, 22–25]. Given the wide variety of diagnostic and treatment modalities for IPH, we surveyed providers to elicit commonalities in management and determine IPH morbidity and mortality.

## Methods

Pediatricians and pediatric subspecialists were surveyed internationally using pediatric pulmonology and rheumatology subspecialty list serves. The web-based survey contained 27 questions. Providers were asked about their practice setting and personal practices for their patients with IPH. Specific questions included details of which diagnostic modalities and medications they used for acute and chronic management of alveolar hemorrhage for each of their patients. Lastly, respondents were given the opportunity to write in comments not addressed in the survey. Morbidity was assessed by time to diagnosis, misdiagnoses and hospitalization rate per patient. Mortality was calculated based on the number of patient deaths before the age of 21 years. We used standard descriptive statistics to describe our results such as percentages, means with standard deviations for parametric data, and medians with interquartile ranges (IQR) for non-parametric data. Percentages were calculated based on the total number of responses per question.

## Results

Eighty-eight providers responded to the survey, representing 274 IPH patients. Respondents saw 0–20 patients per physician in their clinic (median = 3, IQR 1 to 4). Most respondents were pediatric pulmonologists (*n* = 73, 83.0 %). Respondents were in practice for 1 to 51 years (median = 13, IQR 3 to 26). Most respondents practiced in an academic (*n* = 80, 94.1 %) and urban setting (*n* = 74, 85.1 %). Respondents were from five continents representing 15 countries, most being from the United States (*n* = 63, 72.4 %) (Table 1).

**Table 1:** Physician respondent characteristics

Characteristics	n (%)
Specialty, *n* = 88	
Pediatric pulmonologist	73 (83)
Pediatric rheumatologist	13 (14.8)
Pediatrician	1 (1.1)
Allergist	1 (1.1)
Practice location, *n* = 87	
North America	69 (79.3)
Middle East	6 (6.9)
Europe	5 (5.7)
South America	3 (3.4)
Asia	2 (2.3)
Africa	2 (2.3)

Most patients were diagnosed more than 2 months after presentation (*n* = 112, 47.7 %) with 14.5 % (*n* = 34) of patients taking longer than 6 months to diagnosis. Most respondents (*n* = 50, 63.3 %) reported that their patients were initially misdiagnosed.

Misdiagnoses included anemia (*n* = 33, 60.0 %), gastrointestinal bleeding (*n* = 10, 18.2 %), pneumonia (*n* = 6, 10.9 %), and asthma (*n* = 3, 5.5 %). Other misdiagnoses included tachypnea/hemoptysis, pulmonary infarct and upper respiratory tract infection reported by one physician each.

Since IPH is a diagnosis of exclusion, providers assessed for rheumatologic disease (*n* = 83, 97.6 %), other pulmonary disease (*n* = 75, 88.2 %), cardiac disease (*n* = 71, 83.5 %), infection (*n* = 69, 81.2 %), milk protein allergy (*n* = 54, 63.5 %), and celiac disease (*n* = 31, 36.5 %). Milk protein allergy (*n* = 35, 12.8 %), trisomy 21 (*n* = 18, 6.6 %), and celiac disease (*n* = 13, 4.7 %) were comorbidities reported. Written responses for comorbidities by four or less respondents included mild P-ANCA positivity, lupus, hereditary spherocytosis, chronic granulomatous disease, pulmonary arteriovenous malformation, foreign body, asthma, tree nut allergy, autoimmune hepatitis, developmental delay, prematurity, small stature, and metabolic disorder.

Diagnostic methods used by providers included bronchoalveolar lavage (*n* = 72, 91.1 %), chest CT (*n* = 60, 75.9 %), chest radiography (*n* = 54, 68.4 %), hemoglobin and reticulocyte count (*n* = 55, 69.6 %), and lung biopsy (*n* = 41, 51.9 %). Twenty-four combinations of these diagnostic methods were reported. The most common 70.9 % of responses are described in Table 2. The two most frequent combinations included bronchoalveolar lavage, chest CT, chest radiography, and hemoglobin and reticulocyte count (*n* = 37, 46.9 %). Of that, respondents differed in the utilization of lung biopsy (lung biopsy: *n* = 21, 56.8 % versus without lung biopsy: *n* = 16, 43.2 %).

**Table 2:** Evaluations performed to diagnose IPH^†^

BAL	Chest CT	CXR	H&R	Biopsy	Other	n (%)
+	+	+	+	+	+ ^a^	21 (26.6)
+	+	+	+		+ ^b^	16 (20.3)
+	+	+				5 (6.3)
+	+		+	+		5 (6.3)
+		+	+			5 (6.3)
+						4 (5.1)

^a^other included echocardiogram, IgE, PFTs, and/or negative autoimmune labs

^b^other included transfer factor, and/or negative autoimmune labs

†Responses less than 5 % are not shown

Respondents were asked about which medications were used for initial presentation, maintenance, and exacerbation of IPH (Table 3). On initial presentation, most respondents used corticosteroids (*n* = 74, 98.7 %) and 61.3 % (*n* = 46) of those respondents used corticosteroids alone. In addition to corticosteroids, HCQ (*n* = 25, 33.3 %), AZA (*n* = 6, 8.0 %), and CYC (*n* = 3, 4.0 %) were used for initial presentation. The most common medications respondents used for maintenance therapy were corticosteroids (*n* = 63, 84.0 %), HCQ (*n* = 48, 64.0 %), AZA (*n* = 28, 37.3 %), and CYC (*n* = 12, 16.0 %). For IPH exacerbation, all respondents agreed on the use of corticosteroids (*n* = 72, 100.0 %).

**Table 3:** Treatment medications used for initial presentation, maintenance and exacerbations^†^

Initial Presentation					
Steroids	HCQ	CYC	AZA	IVIG	n (%)
+					46 (61.3)
+	+				15 (20)
+	+	+			4 (5.3)
Maintenance					
Steroids	HCQ	CYC	AZA	IVIG	n (%)
+	+				27 (36.0)
+					9 (12.0)
+	+		+		7 (9.3)
+			+		6 (8.0)
			+		4 (5.3)
+	+	+	+		4 (5.3)
Exacerbations					
Steroids	HCQ	CYC	AZA	IVIG	n (%)
+					55 (76.4)
+				+	17 (23.6)

Steroid dosage and frequency for an IPH exacerbation was queried as a free text response. Of the 59 respondents, 69.5 % (*n* = 41) used 0.5–8 mg/kg/day of prednisone, prednisolone, or methylprednisolone for 1–4 weeks followed by a taper, while 30.5 % (*n* = 18) used methylprednisolone 10–30 mg/kg/dose given for 1–3 consecutive days at weekly or monthly intervals.

Most patients were either not readmitted over the course of a year (*n* = 69, 30.4 %) or had 1–2 admissions per year (*n* = 121, 53.3 %) for an IPH exacerbation. Thirty-seven (16.3 %) patients had more than three admissions per year for alveolar hemorrhage. Of the 67 physicians who responded to the question, representing 247 patients, 18 (7.3 %) patients died before 21 years of age.

## Discussion

Though IPH is a rare disorder, we were able to obtain responses from providers internationally caring for 274 patients, one of the largest IPH cohorts to date. We found a mortality of 7.3 %, which is lower than the 16 to 60 % mortality previously reported [3, 6, 7, 25–27]. Physician responses highlighted the commonalities and differences in the management of IPH.

For diagnosis, respondents agreed on the exclusion of rheumatologic disease and obtaining a bronchoalveolar lavage, chest CT, chest radiography, hemoglobin and reticulocyte count. Respondents were divided on whether to perform lung biopsy which can assess for other etiologies of diffuse alveolar hemorrhage like capillaritis. Identifying capillaritis is important because it requires an aggressive treatment regimen early on to avoid serious outcomes [25].

We speculate that respondents who did not pursue lung biopsy relied on autoimmune serology results to exclude capillaritis and diagnose IPH. Case series on diffuse alveolar hemorrhage showed that some patients had negative autoimmune serologies at initial presentation that later became positive; this indicates that serologies at diagnosis are insufficient to diagnose capillaritis versus IPH [4, 7, 28–31]. This is reinforced by Fan and colleagues who found that more than a third of their diffuse alveolar hemorrhage patients had negative autoimmune serologies, but had positive lung biopsies for capillaritis [26, 32]. These studies suggest that lung biopsy is essential to exclude capillaritis in the absence of positive autoimmune serologies and to exclude other disease processes such as infection or other forms of interstitial lung disease requiring different interventions [14].

Respondents agreed on the use of corticosteroids for treatment at initial presentation and for exacerbations. The responses are consistent with Godfrey’s review of 83 published IPH cases, where 92.0 % of patients received corticosteroids for initial treatment with 61.0 % of those patients having no other therapies recorded at initiation.

The steroid regimens included methylprednisolone 2–4 mg/kg/day or 30 mg/kg/dose monthly followed by a steroid wean to a dose as low as prednisolone 0.5–1 mg/kg every other day [27]. For exacerbation, the use of intermittent methylprednisolone as a means to decrease the side effects of daily corticosteroids and IVIG are common amongst our respondents; however, the data for both regimens in patients with alveolar hemorrhage is scant. Most studies are in patients with other rheumatologic disorders and immune mediated renal abnormalities [25, 33–38].

We found that corticosteroids in combination with HCQ, AZA, or CYC were the most common regimens for maintenance therapy amongst our respondents. In 2004, European Respiratory Society Task Force found that corticosteroids (80.2 %, prednisolone 1–2 mg/kg/day) and HCQ (32.8 %, 6–10 mg/kg/day dosed twice daily) were the regimens most used for both pediatric and adult interstitial lung disease. 73.9 % of the ERS respondents reported a good to partial response to corticosteroids and HCQ. Pulmonary hemosiderosis was the fourth most common diagnosis in their patient population [39].

CYC and AZA were also used by our respondents. CYC was demonstrated to be lifesaving for adults with vasculitis and alveolar hemorrhage in the 1970’s despite toxicities such as hemorrhagic cystitis, infertility, malignancy and pulmonary fibrosis [40]. Due to the toxicity profile for CYC, alternative maintenance therapies with better side effect profiles are currently under investigation. A favorable response was noted in adult clinical drug trials such as CYCAZAREM. The CYCAZAREM study conducted by the European Vasculitis Study Group described the utility of AZA for maintenance medication after induction with CYC. Additional trials like the REMAIN drug trial looking at low dose corticosteroids versus AZA will be of particular interest for pediatric physicians heavily dependent on corticosteroids [3, 20, 22, 25, 39–41]. 6-mercaptopurine (6MP) is a thiopurine like AZA with AZA being a prodrug of 6MP [42]; although none of the providers in our survey reported using six mercaptopurine, it has been used as an IPH maintenance therapy by Luo et al. [24]. It is unclear if CYC or AZA/6MP is as effective in children. Furthermore, side effects of each therapy may vary in different patients, which is important in determining if the patient will tolerate the therapy long term. Clinical studies in inflammatory bowel disease revealed a subset of patients have more side effects, such as leukopenia, to thiopurines (AZA/6MP) thought to be due to lack of thiopurine methyltransferase activity, an important enzyme in thiopurine metabolism [24, 42–44]. Therefore, more research needs to be done in the side effects of therapies for patients with alveolar hemorrhage as well.

A major limitation of our survey method was recall bias. Respondents possibly did not report all their patients, misreported patient information, or provided incomplete data sets. Thus, physician management could not be directly correlated with patient outcomes.

## Conclusion

Our study revealed commonalities and disparities in IPH care. In practice, half of our respondents avoided the performance of lung biopsy. Corticosteroids remained the mainstay for treatment even in light of available immunomodulators. These trends point towards the need to standardize IPH care. Establishing a diffuse alveolar hemorrhage registry that links diagnostic and treatment regimens to patient specific outcomes will be necessary to improve IPH care. Improved morbidity and mortality for this rare and grave disease may be achieved by diagnostic algorithms that outline the indications for a lung biopsy and efficacy trials for steroid sparing agents.
